# Delayed Strangulated Hiatal Hernia Post-gastrectomy Masquerading as Diaphragmatic Hernia: A Diagnostic Pitfall

**DOI:** 10.7759/cureus.83274

**Published:** 2025-04-30

**Authors:** Mardiana Mardan, Wei Keat Ooi, Arif Hameed Sultan, Guo Hou Loo, Nik Ritza Kosai

**Affiliations:** 1 Department of Surgery, Universiti Malaya Medical Centre, Kuala Lumpur, MYS; 2 Department of Surgery, Queen Elizabeth Hospital, Kota Kinabalu, MYS; 3 Department of Surgery, Universiti Malaysia Sabah, Kota Kinabalu, MYS; 4 Upper GI and Metabolic Surgery Unit, Department of Surgery, Universiti Kebangsaan Malaysia Medical Centre, Kuala Lumpur, MYS; 5 Upper GI and Metabolic Surgery Unit, Department of Surgery, Hospital Canselor Tuanku Muhriz, Universiti Kebangsaan Malaysia, Kuala Lumpur, MYS

**Keywords:** diaphragmatic hernia, gastroesophageal junction cancer, siewert iii adenocarcinoma, strangulation, surgical complication, total gastrectomy

## Abstract

Strangulated diaphragmatic hernia (DH) is an uncommon but life-threatening complication that can occur years following major upper gastrointestinal (GI) surgery. Its delayed presentation often leads to diagnostic delays and increased morbidity. Unlike common traumatic or congenital diaphragmatic hernias, this case illustrates a delayed hiatal herniation secondary to postoperative anatomical alteration and lack of crural repair after total gastrectomy.

We report a 36-year-old man who developed acute epigastric pain and vomiting two years after undergoing total gastrectomy with Roux-en-Y reconstruction for Siewert III gastroesophageal junction adenocarcinoma. Imaging revealed a strangulated left diaphragmatic hernia with herniation of the Roux and biliopancreatic limbs, including the oesophagojejunostomy. Emergency surgery required conversion to a thoracoabdominal approach for safe reduction and repair. The patient recovered uneventfully.

Delayed DH should be considered in patients with a history of hiatal dissection presenting with acute symptoms. Early cross-sectional imaging and prompt surgical intervention are essential. Prophylactic crural closure during initial surgery may reduce future hernia risk.

## Introduction

Total gastrectomy with D2 lymphadenectomy and Roux-en-Y reconstruction remains the cornerstone treatment for gastric and gastroesophageal junction (GOJ) cancers, particularly Siewert III adenocarcinomas [1,2]. This procedure often involves hiatal dissection, which can predispose to postoperative diaphragmatic hernias - a rare but potentially fatal complication.

The clinical presentation of diaphragmatic hernia (DH) may range from asymptomatic to life-threatening bowel strangulation, necessitating a high index of suspicion and urgent intervention. Grimes (1974) reported that in a series of 980 cases, DH was diagnosed preoperatively in only 43.5% of patients, while 41.3% were diagnosed intraoperatively or at autopsy and the remaining 14.6% had delayed diagnosis after clinical deterioration [3]. Right-sided ruptures were more commonly missed initially. 

This report presents a unique case of herniation occurring via the esophageal hiatus - rather than through a true diaphragmatic defect - as a delayed complication of total gastrectomy, distinct from the more common traumatic or congenital origins, and further explores the surgical nuances, tumour biology and preventive considerations.

## Case presentation

A 36-year-old man presented in March 2021 with a one-year history of intermittent epigastric discomfort, postprandial vomiting and bloating. Upper gastrointestinal (GI) endoscopy revealed a fungating tumour at the gastroesophageal junction, extending from 35 to 40 cm from the incisors and arising from the greater curvature. Histopathology confirmed a diagnosis of poorly differentiated tubular adenocarcinoma. Computed tomography (CT) of the thorax, abdomen, and pelvis (TAP) revealed GOJ wall thickening with no evidence of distant metastasis or local invasion. A staging laparoscopy and peritoneal lavage cytology were negative. The multidisciplinary team (MDT) decided on curative-intent treatment using neoadjuvant FLOT (fluorouracil, leucovorin, oxaliplatin, and docetaxel) chemotherapy. Neoadjuvant FLOT chemotherapy is an established regimen for locally advanced gastric or gastroesophageal junction cancers. It aims to induce tumour shrinkage, improve resectability, and increase pathological complete response rates [2].

Chemotherapy was initiated but discontinued after three cycles due to transaminitis and febrile episodes. Despite this, interval imaging showed tumour regression. The patient subsequently underwent open total gastrectomy with D2 lymphadenectomy and Roux-en-Y reconstruction in June 2021. Intraoperative findings included bilateral hiatal dissection and mobilisation of the GOJ without tumour adherence to the crura. Crural repair was not performed. A biliopancreatic limb measuring 30 cm from the duodenojejunal flexure and a Roux limb of 50 cm were created. Final histopathology revealed a T3N2 poorly differentiated adenocarcinoma. He had completed adjuvant chemotherapy by September 2021.

**Figure 1:**
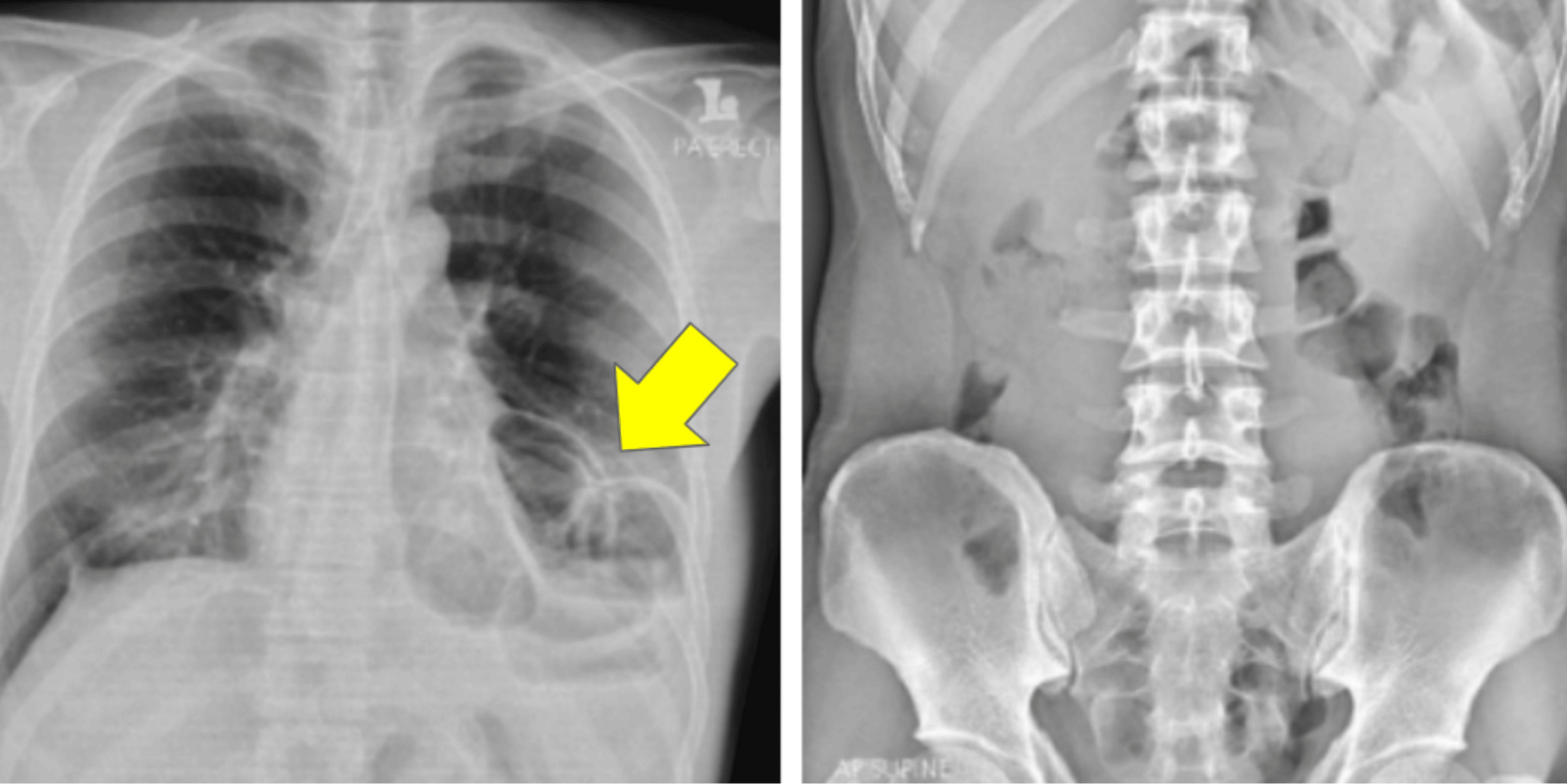
Chest and abdominal radiograph during acute presentation Chest X-ray noted bowel loop at the left hemidiaphragm (yellow arrow). No dilated bowel in the abdominal X-ray.

**Figure 2:**
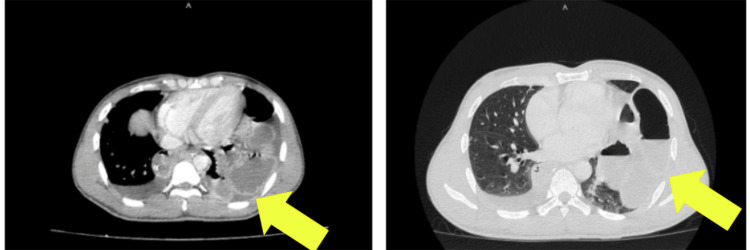
Computed Tomography (CT scan) Thorax, Abdomen and Pelvis - intravenous contrast (right picture), lung window (left picture) Noted the absence of left hemidiaphragm associated with herniation of the bowel (likely dilated jejunum of 5.2 cm in diameter), mesenteric vessels and fat into the left thoracic cavity from the abdominal cavity. There was also mesentery fluid and interloop free fluid adjacent to the herniated bowel. These imaging findings confirmed the left diaphragmatic hernia with signs suggestive of strangulation.

**Figure 3:**
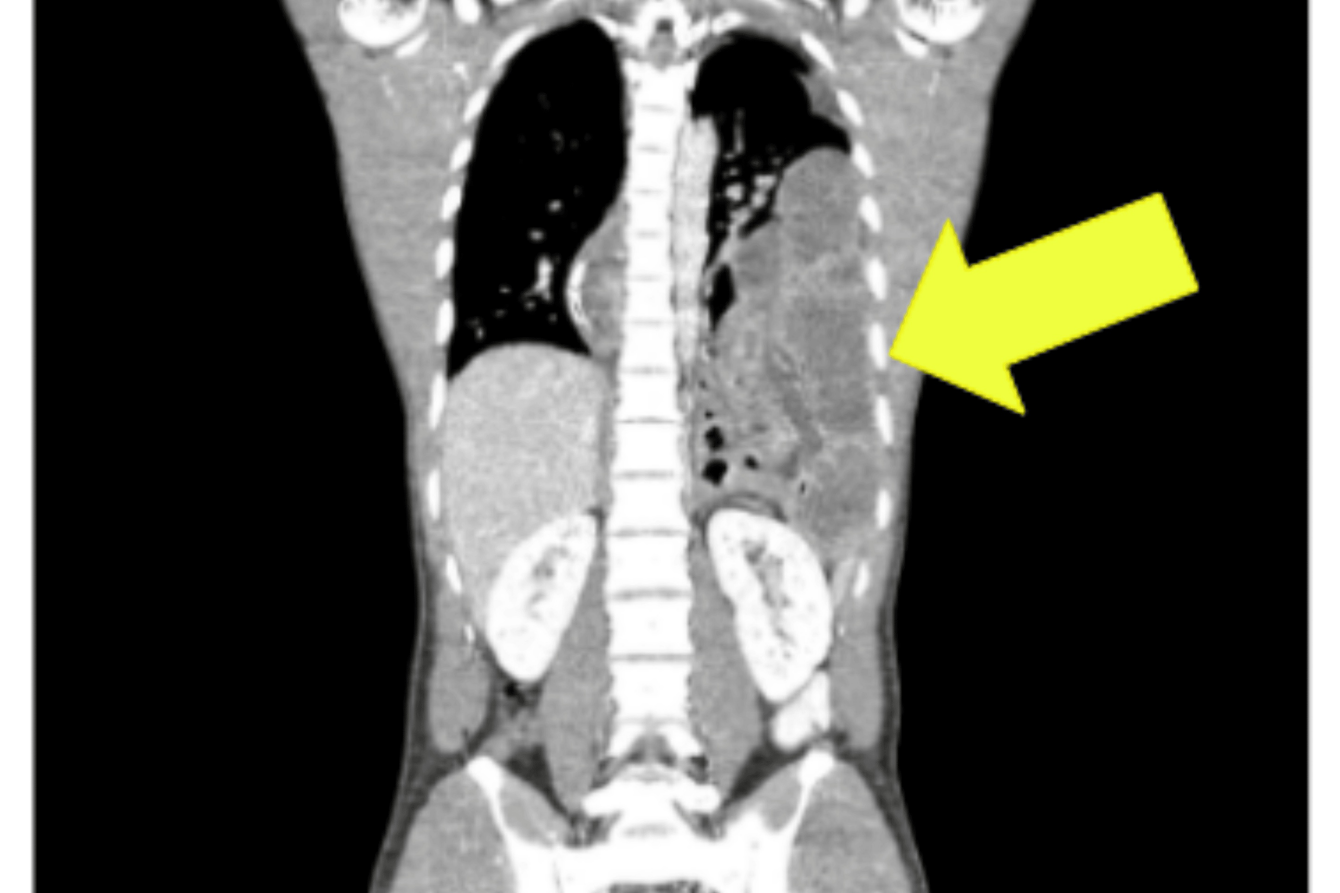
Computed Tomography (CT scan) Thorax, Abdomen and Pelvis - Coronal This accentuates the CT features of the left diaphragmatic hernia with signs suggestive of strangulation (yellow arrow).

In July 2023, the patient presented with a three-day history of epigastric pain and vomiting. He denied recent trauma or exertion. Examination revealed localized tenderness in the epigastric region with guarding. A chest radiograph showed bowel loops in the left hemithorax (Figure 1) and CT TAP confirmed a left diaphragmatic hernia with herniation of dilated jejunum (5.2 cm), mesentery and fat into the thoracic cavity, suggestive of strangulation (Figures 2, 3).

**Figure 4:**
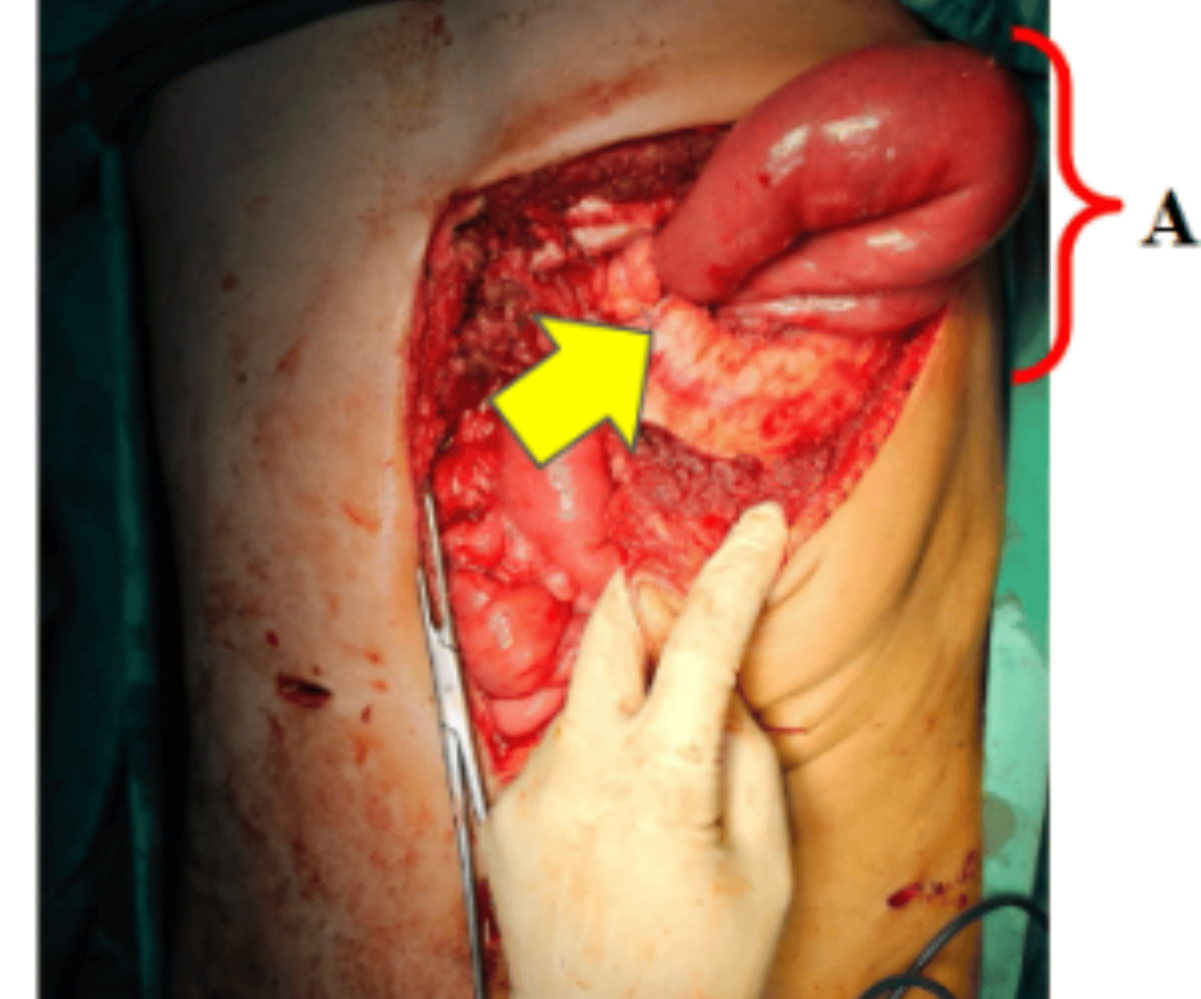
Intraoperative view showing herniation of the Roux limb and previous jejunojejunal anastomosis into the left thoracic cavity through the diaphragmatic defect A: Herniation of the Roux limb and previous jejunojejunal anastomosis. Yellow Arrow: Diaphragmatic defect.

Emergency diagnostic laparoscopy was performed, revealing herniation of the Roux limb, pancreaticobiliary limb, jejunojejunostomy and oesophagojejunostomy through a left diaphragmatic defect (Figure 4).

**Figure 5:**
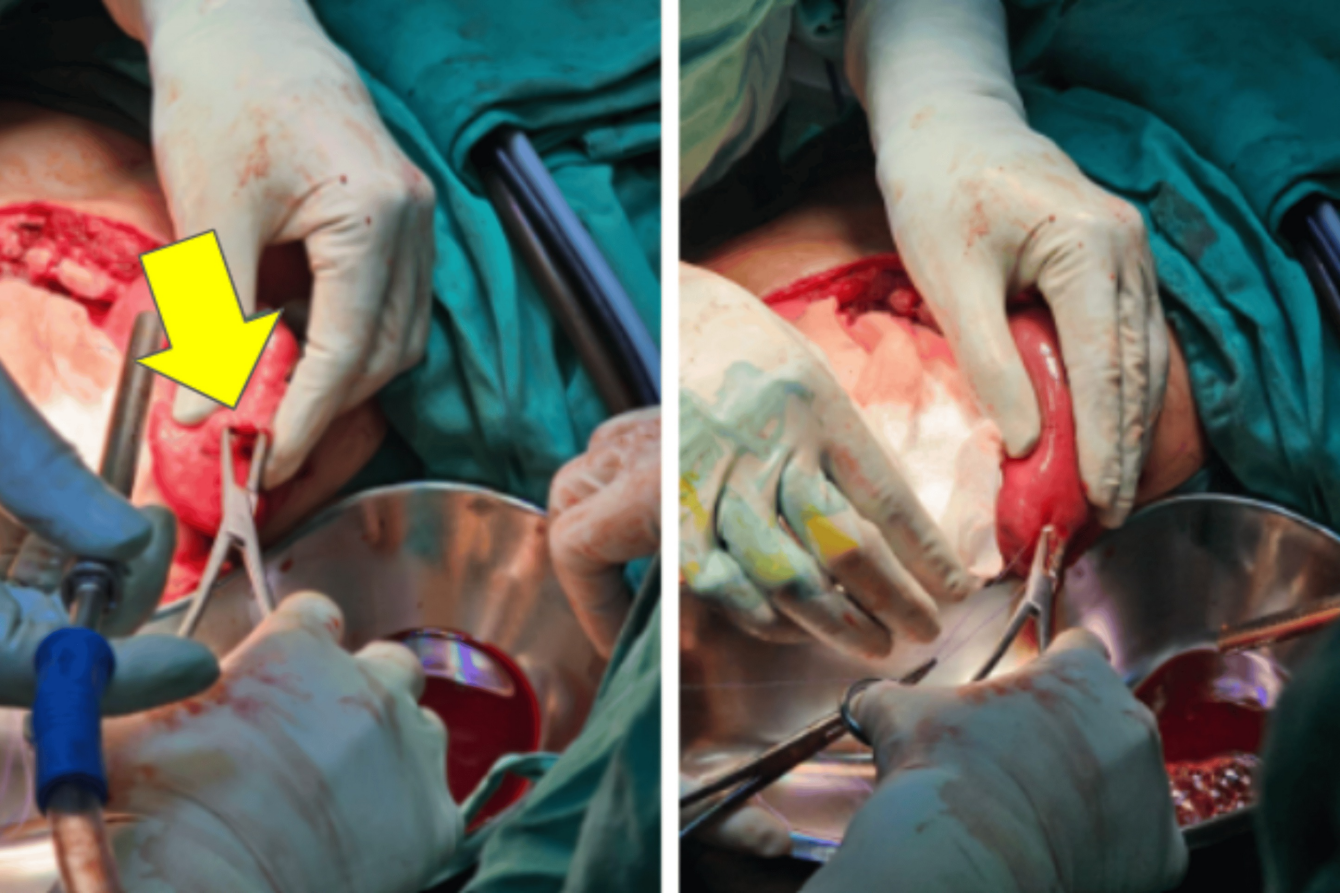
Dilated bowel proximal to the jejunojejunostomy with visible enterotomy site (left picture) and repaired (right picture) Yellow Arrow: Enterotomy site. The enterotomy site was repaired with two-layer absorbable sutures.

Due to dense adhesions and a tight hiatus, conversion to an open left thoracoabdominal approach was necessary. Intraoperative findings included an intact oesophagojejunostomy, a serosal tear and an enterotomy distal to the jejunojejunostomy, both of which were repaired (Figure 5). The serosal tear and enterotomy likely resulted from traction injury during hernia reduction in the setting of dense adhesions and strangulated bowel. These complications, while unintended, are recognized risks during adhesiolysis in emergency surgery. 

**Figure 6:**
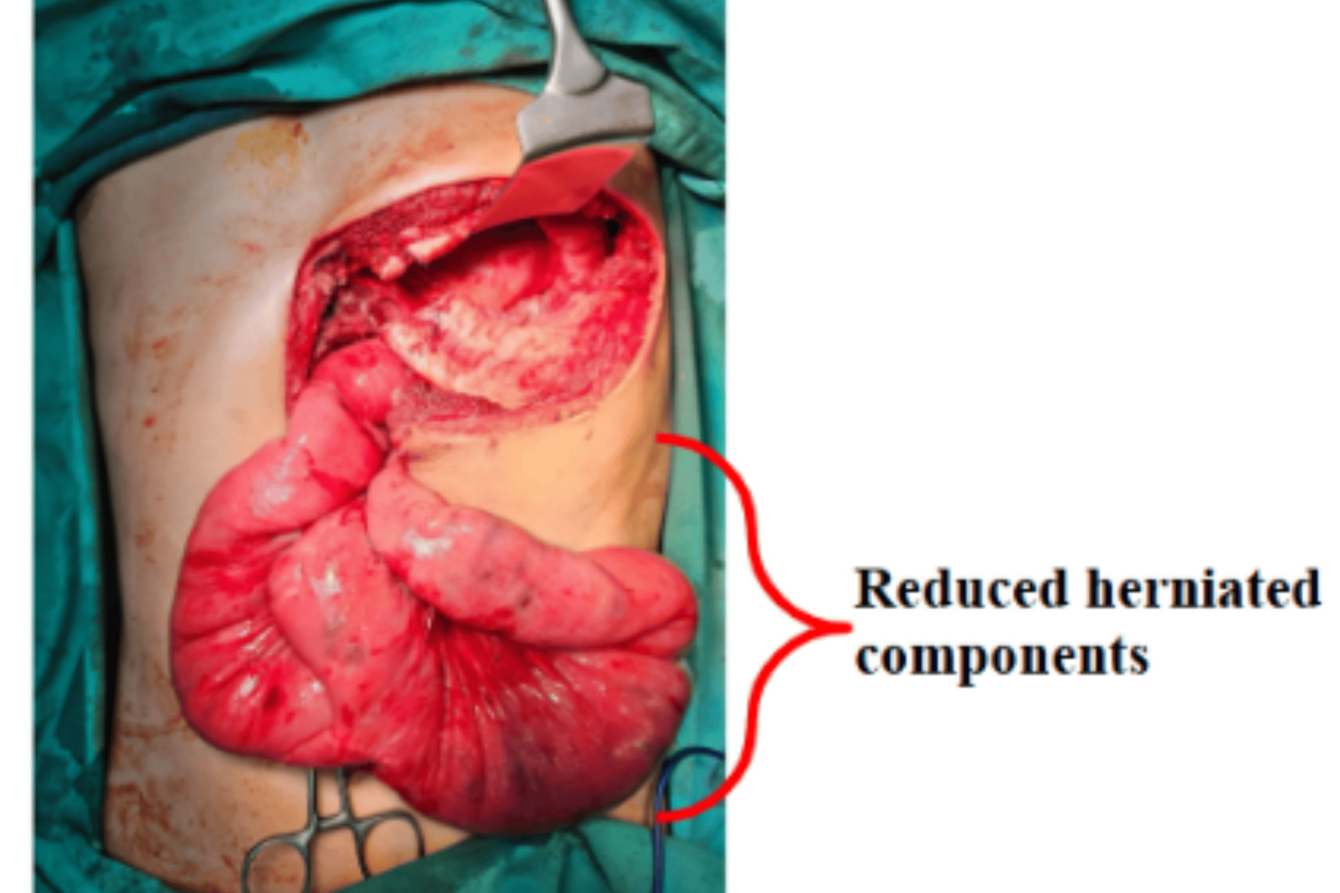
Reduced herniated components into the abdominal cavity following conversion to thoracoabdominal approach

Figure 6 shows the reduced herniated components into the abdominal cavity following conversion to thoracoabdominal approach. The diaphragmatic defect was then closed with interrupted non-absorbable sutures.

**Figure 7:**
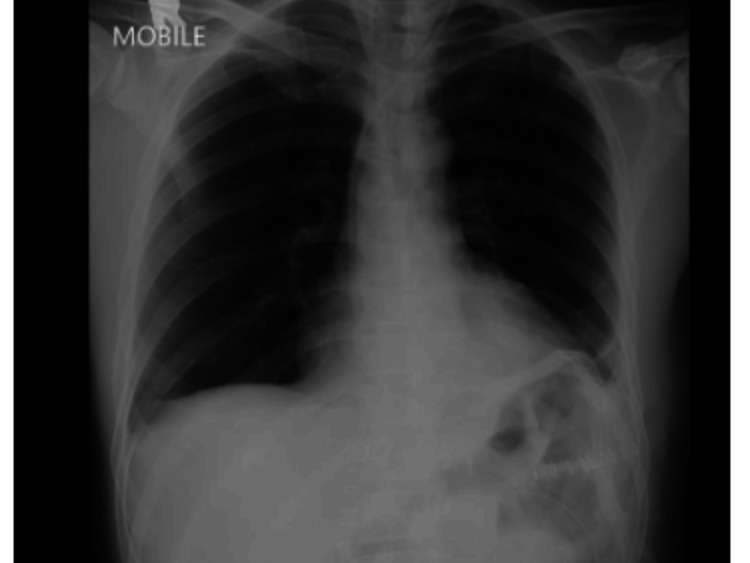
Chest radiograph on post-op day five, prior removing the chest drain and discharge

**Figure 8:**
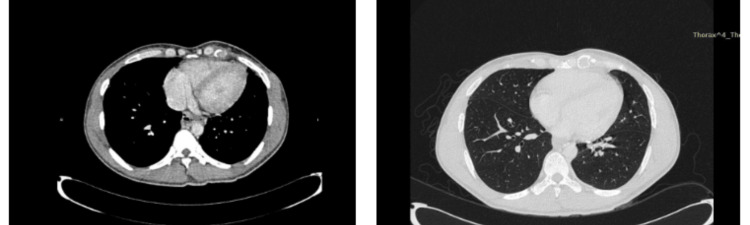
Computed Tomography (CT scan) Thorax, Abdomen and Pelvis on post-op one year No evidence of strangulation or intestinal obstruction.

Postoperative recovery was uneventful. The patient resumed oral intake by postoperative day five (Figure 7) and was discharged on day eight. Follow-up imaging at one year confirmed no recurrence or obstruction (Figure 8).

## Discussion

Total gastrectomy with Roux-en-Y reconstruction remains a mainstay in the management of proximal gastric cancers and Siewert III GOJ tumours. Limb lengths are tailored to minimize bile reflux and optimize nutritional outcomes [4]. Post-gastrectomy complications include common entities such as dumping syndrome, nutritional deficiencies, anastomotic leaks and strictures [5]. Less common but serious complications include internal herniation, hiatal hernia and diaphragmatic hernia [6]. 

While rare, strangulated DH is a serious postoperative complication following upper GI surgery, especially after procedures involving the esophageal hiatus. Although more commonly observed in the context of trauma or congenital defects, this case demonstrates a rare postoperative complication: a herniation via the esophageal hiatus following oncologic upper GI surgery, particularly total gastrectomy with Roux-en-Y reconstruction. The incidence is estimated to be below 2% but is likely underreported due to delayed or subclinical presentations. When strangulation occurs, it becomes a surgical emergency with a risk of ischemia, perforation and mortality if not promptly treated.

In our case, the hernia manifested two years postoperatively in a patient who had undergone open total gastrectomy for Siewert III GOJ adenocarcinoma. While laparoscopic approaches are more commonly associated with postoperative DH due to decreased peritoneal adhesions and increased diaphragmatic mobility [7], our case illustrates that open surgery does not confer complete protection. Importantly, intraoperative findings confirmed that hiatal dissection had been performed, but no crural repair was undertaken - a potentially modifiable risk factor.

Multiple studies have emphasized the role of extensive hiatal dissection, particularly when mobilizing the gastroesophageal junction, in predisposing patients to DH [6,8]. The omission of primary crural closure may allow for progressive widening of the hiatus over time, especially in patients experiencing postoperative weight loss, tissue remodeling or increased negative intrathoracic pressure. In our patient, these factors may have contributed to the gradual development of a hernia that became clinically apparent only upon strangulation.

The herniation of both the Roux limb and the oesophagojejunostomy into the thoracic cavity is particularly notable and rarely described. In some reported series, the herniated contents involve only small bowel or colon; however, in this case, the presence of prior anastomoses within the hernia sac posed significant risks for disruption or leak, necessitating cautious adhesiolysis and eventual conversion to a thoracoabdominal approach for adequate exposure.

Imaging played a pivotal role in diagnosis. While chest radiographs can offer suggestive signs, such as air-fluid levels or bowel loops in the thorax, they are neither sensitive nor specific [9]. Cross-sectional imaging, particularly CT with contrast, remains the gold standard, as it allows for clear identification of the herniated contents, the size of the diaphragmatic defect, signs of strangulation, and potential ischemia. In our patient, CT findings of bowel wall thickening, mesenteric vessel distortion and interloop fluid prompted immediate surgical exploration - a decision that likely prevented irreversible ischemic injury. 

Surgical management must be tailored to the individual case. Although minimally invasive approaches have been successful in selected patients, the presence of dense adhesions, prior anastomoses and anatomical distortion may necessitate conversion to open surgery, as occurred in our case. The repair of the diaphragmatic defect should be tension-free and durable; we opted for non-absorbable interrupted sutures, although mesh reinforcement can be considered in larger defects or recurrences [10]. In selected cases, particularly those involving large hiatal defects or recurrent herniation, mesh reinforcement of the crural repair may be considered to enhance the durability of the repair. A systematic review of 18 studies involving 1,846 patients demonstrated that the use of biosynthetic meshes was associated with reduced recurrence rates, ranging between 0.9% and 25%, and a favorable safety profile with low rates of mesh-related complications [11]. However, a long-term randomized clinical trial with 13 years of follow-up found no significant difference in hernia recurrence rates between non-absorbable mesh reinforcement and suture-only repair, but reported a higher incidence of postoperative dysphagia in the mesh group [12]. Therefore, the decision to employ mesh should be individualized, balancing the potential benefits of reinforcement against the risk of postoperative complications.

Preventive strategies are increasingly advocated, particularly in high-risk patients undergoing extensive hiatal mobilization. Several authors suggest routine crural closure, even in the absence of intraoperative herniation, to prevent delayed DH formation. Others propose assessing the hiatal diameter intraoperatively to guide the decision. However, the optimal technique, whether via posterior crural suturing, anterior reinforcement, or mesh augmentation, remains under investigation.

To contextualize our findings, we reviewed published reports of diaphragmatic hernia following total gastrectomy (Table 1). While the rarity of delayed diaphragmatic hernia post-gastrectomy limits the availability of large-scale data, several case reports and small series have documented similar complications. These studies consistently highlight key risk factors such as hiatal dissection, absence of crural closure and minimally invasive surgical approaches.

**Table 1:** Summarizes selected reported cases, comparing their presentation, surgical details and outcomes with the current case DH: diaphragmatic hernia

Author (Year)	Approach	Timing of DH	Herniated Contents	Risk Factors Identified	Management
Kori et al. (2022) [9]	Laparoscopic	4 months	Small bowel	Hiatal mobilisation. No crural repair	Laparoscopic repair
Gong et al. (2019) [6]	Laparoscopic	4-12 months	Small bowel	Laparoscopic approach. Hiatal dissection	Laparoscopic repair
Urabe et al. (2019) [7]	Laparoscopic	6 months	Jejunum; Colon	Weight loss. Crura not closed	Open repair
Current case	Open	2 years	Roux limb; Biliopancreatic limb	Hiatal dissection. No crural repair	Thoracoabdominal repair

This case highlights the importance of long-term vigilance following major upper GI surgery. Even in asymptomatic patients, awareness of potential late complications such as DH is critical. According to the Society of American Gastrointestinal and Endoscopic Surgeons (SAGES) guidelines, routine imaging surveillance is not necessary for asymptomatic patients; however, patients should be educated to recognize symptoms such as dysphagia, chest pain, or vomiting, which may suggest hernia recurrence or complications [13]. Prompt evaluation of any new gastrointestinal or respiratory symptoms is advised, along with lifestyle modifications to minimize intra-abdominal pressure and reduce recurrence risk.

Prognosis following surgical repair is favorable, provided intervention occurs before irreversible ischemia develops. Nonetheless, recurrence has been reported, and preventive strategies are crucial. Prophylactic crural repair during initial surgery, particularly in patients with large hiatus or extensive mobilisation of the oesophagogastric junction, may reduce the incidence of this complication [10].

The key learning points to be taken from this case are summarized in Table 2.

**Table 2:** Key Learning Points CT TAP: computed tomography of the thorax, abdomen, and pelvis; DH: diaphragmatic hernia

NO	Key Learning Points
1	Delayed diaphragmatic hernia is a rare but life-threatening complication following total gastrectomy, even years after surgery.
2	Hiatal dissection without crural closure may predispose to herniation, especially with postoperative weight loss or altered intrathoracic pressure.
3	Cross-sectional imaging (CT TAP) is essential for accurate diagnosis and surgical planning in suspected strangulated DH.
4	Surgical repair may require conversion to open thoracoabdominal approach for safe reduction, especially when prior anastomoses are involved.
5	Preventive crural repair during the index operation may reduce the risk of delayed DH formation and should be considered routinely.

## Conclusions

Strangulated diaphragmatic hernia is a rare yet life-threatening complication that may present years after total gastrectomy. This case emphasizes the importance of early imaging in at-risk patients, especially those with prior hiatal dissection. Early imaging, a high index of suspicion, prompt surgical intervention and individualized operative planning are critical to prevent morbidity. Crural closure at index surgery should be considered standard in patients with significant hiatal mobilisation. Long-term clinical vigilance remains essential even in asymptomatic survivors of gastric cancer surgery.
